# Collagen Hybrid Formulations for the 3D Printing of Nanostructured Bone Scaffolds: An Optimized Genipin-Crosslinking Strategy

**DOI:** 10.3390/nano10091681

**Published:** 2020-08-27

**Authors:** Giorgia Montalbano, Giorgia Borciani, Giorgia Cerqueni, Caterina Licini, Federica Banche-Niclot, Davide Janner, Stefania Sola, Sonia Fiorilli, Monica Mattioli-Belmonte, Gabriela Ciapetti, Chiara Vitale-Brovarone

**Affiliations:** 1 Department of Applied Science and Technology, Politecnico di Torino, Corso Duca degli Abruzzi 24, 10129 Torino, Italy; giorgia.montalbano@polito.it (G.M.); giorgia.borciani@polito.it (G.B.); caterina.licini@polito.it (C.L.); federica.banche@polito.it (F.B.-N.); davide.janner@polito.it (D.J.); stefania.sola@polito.it (S.S.); sonia.fiorilli@polito.it (S.F.); 2 Scienze e Tecnologie Biomediche, IRCCS Istituto Ortopedico Rizzoli, Via di Barbiano 1/10, 40136 Bologna, Italy; gabriela.ciapetti@ior.it; 3 Department of Clinical and Molecular Sciences (DISCLIMO,) Università Politecnica delle Marche, Via Tronto 10/a, 60126 Ancona, Italy; g.cerqueni@pm.univpm.it (G.C.); m.mattioli@univpm.it (M.M.-B.)

**Keywords:** mesoporous bioactive glasses, type I collagen, genipin, collagen chemical crosslinking, strontium release, osteoblast-like cell, biomimetic composites, bone-tissue engineering

## Abstract

Bone-tissue regeneration induced by biomimetic bioactive materials is the most promising approach alternative to the clinical ones used to treat bone loss caused by trauma or diseases such as osteoporosis. The goal is to design nanostructured bioactive constructs able to reproduce the physiological environment: By mimicking the natural features of bone tissue, the cell behavior during the regeneration process may be addressed. At present, 3D-printing technologies are the only techniques able to design complex structures avoiding constraints of final shape and porosity. However, this type of biofabrication requires complex optimization of biomaterial formulations in terms of specific rheological and mechanical properties while preserving high biocompatibility. In this work, we combined nano-sized mesoporous bioactive glasses enriched with strontium ions with type I collagen, to formulate a bioactive ink for 3D-printing technologies. Moreover, to avoid the premature release of strontium ions within the crosslinking medium and to significantly increase the material mechanical and thermal stability, we applied an optimized chemical treatment using ethanol-dissolved genipin solutions. The high biocompatibility of the hybrid system was confirmed by using MG-63 and Saos-2 osteoblast-like cell lines, further highlighting the great potential of the innovative nanocomposite for the design of bone-like scaffolds.

## 1. Introduction

Nowadays, bone defects and fractures represent one of the most relevant clinical problems, with data reporting more than two million bone grafts per year [1,2,3]. Considering the significant drawbacks and limitations shown by the gold-standard treatments, and the ever-increasing demand for bone substitutes, nowadays tissue-engineering therapies are considered a promising alternative strategy for bone reconstitution [2,3,4,5].

Especially in diseases such as osteoporosis, the design of biomaterials able to direct and promote bone tissue remodeling/regeneration rather than simply filling the bone defect and supporting the damaged tissue is considered a crucial aspect [2,3,6]. In this frame, a regeneration strategy based on biomimetic and bioactive materials that actively interact with the surrounding living tissues holds great potential for a more effective clinical treatment, at variance with the conventional passive biomaterials which often prevent any potential crosstalk with the body tissues [5,7,8]. The proper selection of biologically inspired biomaterials and fabrication processes is thus considered a key factor to design engineered constructs mimicking the complex nano-, micro- and macro-structures, as well as the natural mechanical and biological properties of the native tissue [6,9]. In the field of bone-tissue engineering (BTE), nanomaterials are exploited to design biomimetic constructs, considering that bone itself is characterized by a perfectly arranged nanocomposite nature [5,10]. However, the reproduction of the complex structure of bone at multiple scale levels, as well as the mimicking of its composition and biochemical cues, still represents a challenging target, and the ideal combination of materials and techniques has not yet been found [11,12].

Based on this, effective bone scaffolds should be designed as osteoconductive and osteoinductive constructs presenting both micro- and nano-architectures, to increase the structural biomimicry while enhancing the ability to stimulate bone regeneration [2,5,11].

Among the bioactive and biomimetic biopolymers, type I collagen, the main organic component of the bone extracellular matrix, is widely known to have distinctive biological properties, due to the high density of amino acid sequences that positively influence cell bio-recognition and adhesion [13]. However, due to the low biomechanical stiffness and the rapid degradation rates shown by in vitro reconstituted collagen matrices [11,14,15], they are often combined with inorganic phases acting as reinforcing agents [16,17,18,19]. In particular, considering the natural composition of bone, hydroxyapatite particles or bioactive glasses are often added to the biomimetic composites as potential inorganic constituents [18,20]. Mesoporous bioactive glasses (MBG) have largely been recognized as effective osteoconductive materials [20,21,22] thanks to their ability to promote the formation of hydroxyapatite crystals, as well as the release of calcium and silicon ions as dissolution products, and to their very high specific surface area, which make them more reactive than traditional ones. More recently, to empower the osteogenic potential of bioactive glasses, several studies have reported a beneficial effect of strontium ions on cell behavior, mainly due to its similarity with calcium, by enhancing new bone formation while reducing osteoclast resorptive activity [21,23,24,25].

The combination with inorganic phases is known to provide enhanced mechanical stiffness and stability to collagen-based matrices, but, by itself, it may be insufficient. In the natural extracellular matrix, collagen molecules are covalently bonded through the action of specific enzymes, ensuring the formation of a stable and stiff matrix composed by a dense fibrillar packing [11,26]. Therefore, to provide the final constructs with improved mechanical features, the formation of covalent bonds between protein molecules has been investigated by exploiting several exogenous crosslinking methods [17].

Considering that some of the most used and efficient collagen crosslinkers, including carbodiimides, formaldehyde and glutaraldehyde, are associated with significant drawbacks such as cytotoxicity and foreign body response, natural agents are preferred to ensure biocompatibility [16]. With this perspective, genipin, a naturally derived chemical crosslinker extracted from gardenia fruit (*Gardenia jasminoides* Ellis), has already shown promising results in terms of enhanced mechanical and enzymatic stability of collagenous matrices and reduced cytotoxic effect [16,27].

The final design of the nanostructured constructs highly depends on a proper combination of materials and manufacturing technology. Conventional techniques are normally based on subtractive methods that limit the precise control over the final morphological features, at variance with 3D printing that has emerged as the most suitable and versatile technology for the fabrication of complex scaffold microarchitectures [28,29,30,31]. Extrusion-based 3D-printing technologies have thus significantly increased the scaffold manufacturing precision, as well as process repeatability and biocompatibility [5,30,31]. Biomaterial printability and high printing fidelity are directly related to the specific viscoelastic properties of the processed materials, with clear requirements to allow for effective extrusion and deposition and eventually for successful optimization of the designed constructs [29,32,33]. These aspects are a critical issue when natural polymers are considered, as they must be treated in a narrow range of thermal and chemical conditions, and they normally require support to avoid structure collapse and deformation while printing [29,34].

To address all the aspects introduced above, the present work is focused on the development of a hybrid biomimetic system suitable for the 3D printing of bone-like scaffolds. The nature of the composite material and the great potential offered by additive manufacturing technologies were simultaneously exploited to design a controlled architecture both at the nano- and micro-scale. Based on this, type I collagen and nano-sized particles of strontium-containing MBG were selected, to obtain a bioactive ink characterized by a sol-to-gel transition upon physiological pH and temperature, with the subsequent reconstitution of a nanostructured fibrillar matrix, resembling the natural fibrillogenesis process occurring in vivo. The biomimicry of the system was boosted considering the ratio and volume percentages of the organic and inorganic components of bone, while the subsequent deposition of hydroxyapatite was favored by the high bioactivity of MBG particles. A comprehensive rheological study of the developed ink was performed to detect the material processability with an extrusion-based printing system. Mesh-like 3D constructs were manufactured while exploiting a supporting bath (according to the freeform reversible embedding of suspended hydrogels (FRESH) method patented by Feinberg A. and Hinton T., in 2016), by means of a commercial BIO X Bioprinter (Cellink) to further prove the system printability [29,35]. The additional ability to release osteoinductive strontium ions was assessed by using ICP analyses and was optimized according to the selected crosslinking method. Due to the weak nature of the material after the physical crosslinking at 37 °C and pH 7.4, we explored the effect of genipin treatment on the hybrid system by measuring the increase in material strength and stability. Furthermore, the protocol for genipin crosslinking was developed to minimize the strontium ion release during the crosslinking process. To this aim, the use of aqueous and ethanol-based solutions as a medium for genipin crosslinking was investigated and compared. The exogenous genipin crosslinking provided strong chemical bonds between collagen molecules that were otherwise lacking in in vitro reconstituted matrices.

The biological assessment was performed on genipin crosslinked samples, using two types of osteoblast-like cells, MG-63 and Saos-2 cell lines, widely reported as a powerful tool in basic and applied biology field, thanks to the greater cell availability, when compared to primary cells, and the consequent reproducibility of results [36,37]. Both are osteosarcoma-derived osteoblasts, but they show different features: MG-63 cells show both mature and immature osteoblastic features, while Saos-2 exhibit a mature osteoblastic phenotype. The use of both cell types allowed an effective investigation of osteoblast behavior following the preliminary assessment of the cell–material interaction [38].

Cell viability was detected after 24 and 72 h from cell seeding onto biomaterial samples, and the cell morphology was observed through Scanning Electron Microscopy (SEM).

## 2. Materials and Methods

### 2.1. Preparation of the Coll/NanoMBG_Sr4% Hybrid System

**Figure 1:**
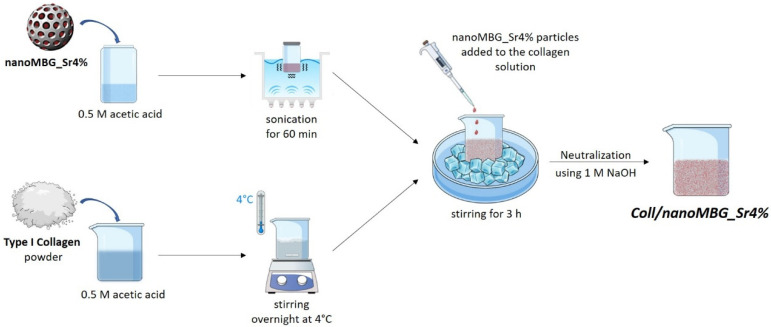
Schematic representation of the preparation of Coll/NanoMBG_Sr4% suspension.

MBG nanoparticles counting 4 mol.% of strontium (Sr/Ca/Si = 4/11/85) were synthesized, following a base-catalyzed sol–gel synthesis previously reported by the authors [21,39]. To promote a homogeneous dispersion into the collagen solution, nano-sized MBG_Sr4% particles (nanoMBG_Sr4%) were previously suspended in 0.5 M acetic acid and further sonicated for 60 min (Digitec DT 103H, Bandelin, Berlin, Germany). The particle suspension was subsequently added to the collagen solution, obtained after dissolving type I collagen powders derived from bovine Achilles tendon (Blafar Ltd., Dublin, Ireland) in the same solvent, as schematically represented in Figure 1. The relative amount of collagen powders and nanoMBG_Sr4% was calculated based on the ratio and volume percentages of the organic and inorganic constituents of bone, where collagen and hydroxyapatite count for 53 vol.% and 47 vol.%, respectively [40]. The resulting suspension of collagen and nanoMBG_Sr4% reached a final collagen concentration of 1.5 wt.% after pH neutralization, using 1 M NaOH. The entire process and the storage were conducted at 4 °C, to prevent premature material gelation.

### 2.2. Morphological and Bioactivity Assessment of Coll/NanoMBG_Sr4%

The morphology and size of nanoMBG_Sr4% were analyzed through Field-Emission Scanning Electron Microscopy (FESEM) with a ZEISS MERLIN instrument (Carl Zeiss AG, Oberkochen, Germany), while the size distribution was further confirmed by dynamic light scattering (DLS), using a Zetasizer Nano ZS90 equipment (Malvern Panalytical, Malvern., UK), after dispersing the particles in 0.5 M acetic acid at the same concentration considered for the hybrid suspension. Tests were performed in triplicate, at room temperature.

Morphological and bioactive properties of the hybrid system were investigated following the gelation of Coll/NanoMBG_Sr4% triggered by physiological temperature (37 °C) and pH (7.4). Samples of physically crosslinked Coll/NanoMBG_Sr4% were obtained by pipetting the hybrid suspension in a silicon mold that was subsequently incubated at 37 °C for 3 h. The collected bulk samples were then stored at 4 °C until testing.

For morphological analyses, material samples were lyophilized for 24 h after freezing at −20 °C, using a Lyovapor L-200 freeze-dryer (Büchi, Flawil, CH) under vacuum (<0.1 mbar). Cross-sections of lyophilized samples were sputter-coated with platinum (7 nm thickness layer) and analyzed by FESEM.

Based on procedures previously reported in the literature, the in vitro formation of hydroxyapatite crystals was observed after soaking Coll/NanoMBG_Sr4% samples in 3 mL of Simulated Body Fluid (SBF) at 37 °C [41,42]. At each time point (1, 3 and 7 days), three samples were collected, washed in distilled water (dH_2_O) for 12 h and lyophilized before FESEM and XRD analyses, to evaluate the apatite crystal precipitation.

### 2.3. Printability of Coll/NanoMBG_Sr4% Suspension

Rheological tests on the Coll/NanoMBG_Sr4% suspension were performed, to investigate its viscoelastic character and its potential as material ink for the 3D printing of high-resolution constructs. The material printability was preliminary assessed by using a BIO X 3D Bioprinter (Cellink, Gothenburg, Sweden).

#### 2.3.1. Rheological Tests

The viscoelastic properties of the material were investigated by using a DHR-2 controlled stress rotational Rheometer (TA Instruments-New Castle, DE, USA. The instrument was specifically equipped with a 20 mm parallel plate geometry, while the precise and constant control over the environmental conditions was ensured by a Peltier plate system. The suspension viscosity and yield stress were measured at different shear conditions by performing flow ramp tests, at 10 °C, where shear rates values varied between 0.01 and 1000 s^−1^. Furthermore, time-sweep analyses were performed at 37 °C by applying an oscillatory stress at 1% strain and 1 Hz for 60 min to detect the sol–gel transition of the hybrid system.

#### 2.3.2. Preliminary Printing Tests

Mesh-like scaffolds (10 × 10 mm surface, 1 mm thickness) were obtained by using a BIO X Bioprinter, exploiting a temperature-controlled pneumatic print-head. The print-head temperature was set at 10 °C, to keep constant the viscoelastic properties of the material, while 27 G needles (200 μm inner diameter) were used to enhance the final structure resolution. The deposition of the Coll/NanoMBG_Sr4% suspension was supported by a gelatin slurry obtained by following a procedure previously reported in the literature [29] and kept at 20 °C during the entire printing process. In brief, to create the slurry, a 4.5 wt.% gelatin solution was obtained by dissolving porcine gelatin powders (Type A, Sigma-Aldrich, St. Louis, MO, USA) in 300 mL of Dulbecco Phosphate Buffered Saline (D-PBS, Sigma-Aldrich, St. Louis, MO, USA) and gelled at 4 °C overnight. Then, 200 mL of D-PBS at 4 °C was added to the gelled gelatin in a 500 mL jar, and everything was blended for 120 s. The gelatin suspension was transferred into 50 mL conical tubes and centrifuged (3800 rcf, 4 min), to collect the slurry particles, after accurately removing the supernatant. The collected gelatin slurry was then stored at 4 °C until use.

Printing speed, air pressure and layer height were selected to obtain the best resolution according to the considered mesh-like 3D model.

After printing, scaffolds were incubated at 37 °C, to promote the material gelation, while also enabling the removal of the gelatin bath.

### 2.4. Genipin-Crosslinking in Aqueous and Ethanol-Based Medium

#### 2.4.1. Genipin Crosslinking

**Figure 2:**
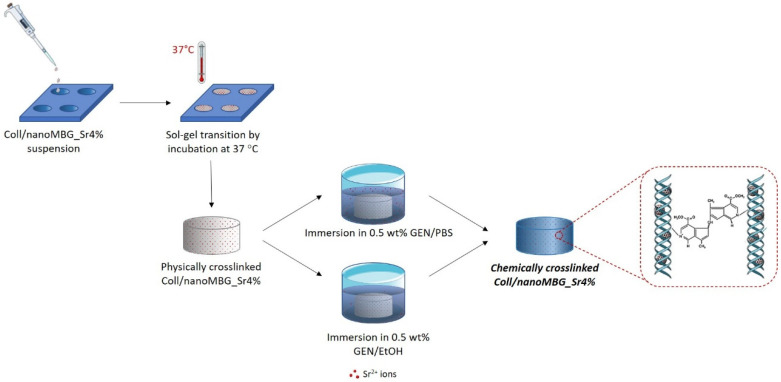
Schematic representation of the chemical crosslinking procedure.

As illustrated in Figure 2, two different solutions were prepared by dissolving genipin powders (Challenge Bioproducts, Yun-Lin Hsien, Taiwan) in Dulbecco’ Phosphate Buffered Saline (D-PBS, Sigma-Aldrich, St. Louis, MO, USA) (GEN/PBS) or 70% ethanol (GEN/EtOH). Genipin is used to promote collagen crosslinking and the two different solvents were compared to investigate their effect on strontium ion release that should be avoided during the crosslinking process.

As schematically represented in Figure 2, physically crosslinked samples of Coll/NanoMBG_Sr4% were then chemically crosslinked by immersion in 0.5 wt.% GEN/PBS or 0.5 wt.% GEN/EtOH solutions at 37 °C for 24 h. The samples were then washed two times in dH_2_O, under mild agitation, to remove genipin residues. The formulation and the different chemical crosslinking agents are summarized in Supplementary Materials Table S1.

#### 2.4.2. Strength and Thermal Stability of Crosslinked Coll/NanoMBG_Sr4%

The variation in the viscoelastic properties of the system before and after the chemical crosslinking was investigated by means of rheological tests.

In detail, a dynamic amplitude sweep was carried out varying the percentage of strain between 0.01% and 1% at a constant frequency of 1 Hz and at 37 °C, registering the value of storage (G′) and loss (G″) moduli after both GEN/PBS and GEN/EtOH crosslinking treatment. The same samples underwent an oscillation temperature ramp between 25 and 80 °C, under 1% strain and 1 Hz, using a ramp rate of 5 °C/min to detect the denaturation temperature of the material associated to the sharp decrease of the overall complex modulus (G′, G″).

#### 2.4.3. In Vitro Degradation Tests

The stability of the GEN/PBS and GEN/EtOH crosslinked system was tested by using both hydrolytic and enzymatic degradation tests. The percentage of weight loss was measured after incubation of crosslinked Coll/NanoMBG_Sr4% samples in D-PBS and collagenase solutions at different time points and further compared with that of non-crosslinked samples.

Collagenase from Clostridium histolyticum (type I, Sigma Aldrich, St. Louis, MO, USA) was used for the enzymatic degradation study of both crosslinked and non-crosslinked samples of Coll/NanoMBG_Sr4%. Each sample was immersed in 1 mL of DMEM (Dulbecco’s Modified Eagle’s Medium, Sigma-Aldrich, St. Louis, MO, USA) containing 1 mg of collagenase (2.1 units) and subsequently incubated at 37 °C under mild agitation (50 rpm). At predefined time points (12, 24 and 48 h) samples were collected and abundantly washed in distilled water and then frozen.

D-PBS was selected as the medium for performing hydrolytic degradation of both crosslinked and non-crosslinked samples of Coll/NanoMBG_Sr4%. The samples were immersed in 3 mL of D-PBS, at 37 °C, under mild agitation (50 rpm), collected at predefined time points (24 h, 72 h, 7 days and 14 days) and frozen. These samples were then lyophilized and accurately weighted to record the weight after enzymatic digestion and hydrolytic degradation. After weighting lyophilized non-treated samples, weight loss was assessed by using the following formula:

W= [(W_0_ − W_d_)/W_0_] × 100

where W_0_ represents the initial mass of the sample, W_d_ represents the mass after degradation and W is the resultant loss percentage. Three samples were considered for each time point, and results were reported as mean ± standard deviation.

#### 2.4.4. Strontium Ion Release

To analyze ion release kinetics from the collagen hybrid formulation and the influence of the investigated crosslinking solutions, the concentration of Sr^2+^ was detected up to 14 days of incubation at 37 °C in Tris HCl buffer (Tris(hydroxymethyl)aminomethane (Trizma) (Sigma-Aldrich, St. Louis, MO, USA) 0.1 M, pH 7.4). To this aim, the release of strontium ions was firstly measured during the chemical treatment with GEN/PBS and GEN/EtOH solutions and subsequently by soaking the crosslinked Coll/NanoMBG_Sr4% samples in Tris HCl buffer [21,43,44]. In details, the samples were firstly soaked in 3 mL of 0.5% GEN/PBS or GEN/EtOH solution for 24 h; the crosslinking solution was collected and refreshed at 10 h, while, after 24 h, it was collected and replaced with 3 mL of Tris HCl buffer, that was entirely refreshed at day 3 of incubation. The supernatant collected at the predefined time points (10 h, 24 h, 3 days, 7 days and 14 days) was analyzed by Inductively Coupled Plasma Atomic Emission Spectrometry Technique (ICP-AES) (Thermo Scientific, Massachusetts, USA, ICP-MS, ICAP Q) after proper dilution. The percentage of ion released was calculated by considering the amount of strontium initially incorporated in MBG particles and equivalent to 4% molar percentage, as well as the content of inorganic phase incorporated into the hybrid system. To confirm the theoretical data, samples of the hybrid system were previously dissolved in a mixture of nitric and hydrofluoric acids and subsequently analyzed, via ICP, in order to calculate the final percentage of the released ions starting to the ppm values measured during the analysis.

Analyses were conducted in triplicate, and the percentages of released ions are reported as mean value ± standard deviation.

### 2.5. In Vitro Biological Assessment

#### 2.5.1. MG-63 and Saos-2 Cell Culture

MG-63 human osteosarcoma cells (ATCC, Manassas, VA, USA, CRL-1427) were obtained from Flow Laboratories. The cells were maintained in a standard medium composed of Minimum Essential Medium Eagle-Alpha Modification (α-MEM) (Sigma-Aldrich, St. Louis, MO, USA, M0644), 1% penicillin–streptomycin (Euroclone, Milan, IT, ECB3001D) (penicillin 10.000 Units/mL, and streptomycin 10 mg/mL) and 10% fetal bovine serum (FBS) (Sigma-Aldrich, St. Louis, MO, USA, F7524) in a humidified incubator at 37 °C, 95% air and 5% CO_2_. For passaging trypsin/EDTA (Trypsin 0.05%–EDTA 0.02% in PBS, Euroclone, Milan, IT, ECB3052D) was used.

Saos-2 human osteosarcoma cells (ATCC, Manassas, VA, USA, HTB-8) were purchased from ATCC. Cells were maintained in high/low glucose (1:1) Dulbecco Modified Eagle’s Medium (H-DMEM, Sigma-Aldrich, St. Louis, MO, USA, D6429) (L-DMEM, Euroclone, Milan, IT, ECM0070L), 1% penicillin–streptomycin (Thermo Fisher Scientific, Massachusetts, USA, 15140122) and 10% FBS (Corning Inc., New York, USA, 35-079-CV), without any additional supplement in a humidified incubator at 37 °C, 95% air and 5% CO_2_. For passaging trypsin/EDTA (trypsin 0.05%– EDTA 0.02% in PBS, Sigma-Aldrich, St. Louis, MO, USAT4174) was used.

The medium was refreshed every 3 days for both cell cultures.

#### 2.5.2. MG-63 and Saos-2 Cell Seeding on Coll/NanoMBG_Sr4%

To perform biological tests, we obtained a thin biomaterial layer by deposition of 200 µL of Coll/NanoMBG_Sr4% hybrid suspension in each well of 48-well plates (well surface area ~0.95 cm^2^). The plates were incubated for 3 h at 37 °C and 95% humidity, to promote the sol–gel transition of Coll/NanoMBG_Sr4%. Then, samples were chemically crosslinked with 1 mL of the genipin crosslinking solution, either 0.5% GEN/PBS or 0.5% GEN/EtOH, incubating at 37 °C, 95% humidity for 24 h. To remove any residual of genipin crosslinker, two washing steps with PBS were performed by placing the crosslinked samples in an orbital shaking for 30 min at 37 °C, 95% humidity. Before cell seeding, 2 mL of culture medium were added in each well, to pre-wet the material for 2 h at 37 °C and 95% humidity. 

Cells at 80% of confluence were trypsinized, and MG-63 cells (2 × 10^5^/well) and Saos-2 cells (0.5 × 10^5^/well) were seeded on biomaterial samples, while cells plated at the bottom of tissue culture polystyrene (TCPS) wells were used as controls. The plates were incubated at 37 °C, 95% humidity, and cell adhesion, viability and morphology were assayed at 24 and 72 h after cell seeding.

#### 2.5.3. Cell Viability 

To assess the presence of metabolically active MG-63 and Saos-2 cells seeded onto GEN/EtOH or GEN/PBS crosslinked samples, the one-step Alamar Blue assay (Invitrogen, Carlsbad, CA, USA, DAL1100) was performed according to the manufacturer’s instructions. Briefly, the culture medium was removed, replaced with the Alamar Blue solution prepared as 10% *v/v* in fresh cell culture medium and incubated at 37 °C, 95% humidity for 4 h. Then the fluorescence of Alamar Blue solution was quantified by using a microplate reader (Infinite F200 PRO, TECAN, Mannedorf, Switzerland) at 535 nm excitation and 590 nm emission wavelengths. Data are expressed as a percentage of control cultures (i.e., MG-63 and Saos-2 seeded in tissue culture plates) and reported as mean value ± standard deviation of triplicates. The hybrid material without cells was also analyzed and considered as background.

#### 2.5.4. Morphological Analysis

Cell-seeded samples for SEM analysis were fixed in 2% glutaraldehyde (MERCK, 4239) in 0.1 M sodium cacodylate buffer (Sigma, St. Louis, MO, USA, C-0250), followed by washing in PBS and post-fixation in 1% osmium tetroxide (Electron Microscopy Sciences, Hatfield, PA, USA, 12310) in 0.1 M sodium cacodylate buffer. Complete dehydration was achieved in graded alcohol series and hexamethyldisilane; platinum was used for sputter coating (up to 7 nm thickness). Images were acquired by using a Desktop SEM Phenom XL (Phenom-World B.V., Eindhoven, NL) at an accelerating voltage of 15 kV.

#### 2.5.5. Statistical Analyses

Statistical analyses regarding the differences between the experimental groups and between the two time-endpoints were accomplished by using the nonparametric Mann–Whitney test for unpaired data, using the StatView 5.01 for Windows software (SAS Institute Inc., Cary, NC, USA). Results were considered as mean and transformed into a percentage of viability. Only *p* < 0.05 was considered statistically significant. The *p*-value was at the margin of statistical significance, since it was attested at 0.0495.

## 3. Results

### 3.1. Morphological and Bioactive Properties of the Coll/NanoMBG_Sr4% Hybrid System

Nanoparticles of MBG containing 4 mol.% of strontium were synthesized by following a traditional sol–gel synthesis previously optimized and reported by the authors [21], obtaining a spherical morphology and nano-metric dimension, as illustrated in Supplementary Materials Figure S1A. The inorganic particles showed a monodisperse and narrow size distribution ranging between 100 and 500 nm (Supplementary Materials Figure S1B) with a mean diameter of 237.3 ± 14.71 nm, where the detection of sizes greater than 300 nm was mainly related to the formation of particle agglomerates. The high specific surface area (about 500 m^2^/g), regular mesopores of about 4 nm and pore volume of about 0.52 cm^3^/g were obtained thanks to the use of cetyltrimethylammonium bromide (CTAB) as a templating agent, as reported in a previous study by the authors [39]. Once prepared, nanoMBG_Sr4% were mixed with type I collagen solution (1.5 wt.%) to form a homogeneous hybrid suspension at temperatures lower than 10 °C, subsequently neutralized to enable the reconstitution of a fibrillar matrix in physiological conditions [13,39]. As expected, the subsequent incubation of the suspension at 37 °C promoted the sol–gel transition of the system due to the physical crosslinking of collagen, that was exploited to produce solid samples further lyophilized to perform morphological analyses, using FESEM.

**Figure 3:**
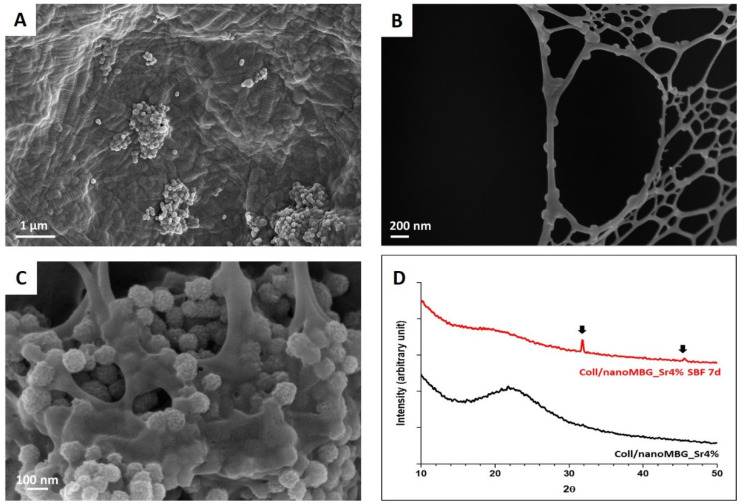
Cross-section FESEM images of Coll/NanoMBG_Sr4% lyophilized samples at different magnifications (**A**,**B**). FESEM image (**C**) and XRD patterns (**D**) of Coll/NanoMBG_Sr4% samples after seven days of incubation in SBF.

Figure 3A,B proves the successful reconstitution of collagen fibers while showing the micro- and nano-architecture resulting from the organization of nanoMBG_Sr4% within the collagenous fibrils and meshes, evidencing the nanoparticles homogeneous embedding into the organic structure. Despite the occurrence of some particle agglomerates, in certain cross-sections, nanoMBG_Sr4% appeared well distributed along the collagen fibrils leading to the formation of hybrid nano-structures, thus mimicking the organization of hydroxyapatite (HA) crystals in the bone extracellular matrix.

The potential bioactivity of the system was investigated by soaking the samples of Coll/NanoMBG_Sr4% in SBF at 37 °C. The presence of HA was observed with FESEM on sample cross-sections, while XRD analyses confirmed the chemical composition of the deposits. At day seven of incubation in SBF, the presence of HA was detected mainly at the surface of MBG particles, as shown in Figure 3C.

The deposition of calcium phosphates was confirmed by XRD analyses, which clearly showed the presence of crystalline peaks of HA superimposed on the broad reflection halo exhibited by Coll/NanoMBG_Sr4%. The detected peaks matched the ones reported by the International Centre for Diffraction Data as a reference for HA [41,45]. As shown in Figure 3D, the mineralization of Coll/NanoMBG_Sr4% samples is confirmed by the appearance of reflection peaks at 31.9° and 46°, where the narrow peak at 31.9° implies the high crystallinity of HA [20].

### 3.2. Printability of Coll/NanoMBG_Sr4% Suspension

To assess the suitability of the Coll/NanoMBG_Sr4% system for extrusion printing applications, its viscoelastic properties were fully investigated by means of rheological characterization. Subsequently, mesh-like 3D-printed structures were obtained through the extrusion-based approach, to identify the optimal printing parameters and conditions when using 27 G needles.

#### 3.2.1. Rheology of Coll/NanoMBG_Sr4% Suspension

**Figure 4:**
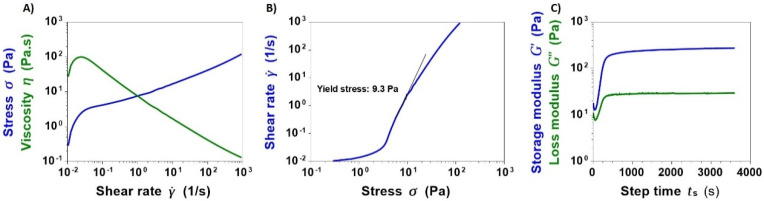
Shear-thinning (**A**), yield stress (**B**) and sol–gel transition (**C**) of Coll/NanoMBG_Sr4% suspension.

Flow ramp tests performed on the Coll/NanoMBG_Sr4% suspension at 10 °C showed the variation of the suspension viscosity as a function of increasing shear rates from 0.01 s^−1^ up to 1000 s^−1^. As illustrated in Figure 4A, the suspension showed a sharp decrease in viscosity down to 0.13 Pa.s with increasing shear rates, proving its pseudo-plastic behavior. Considering low shear rates, the greatest value of viscosity was found to be 99.4 Pa.s. A yield stress value of about 9 Pa was calculated by measuring the onset point corresponding to the change in the curve slope in Figure 4B.

The transition from a liquid-to-solid state of the Coll/NanoMBG_Sr4% system was then monitored by means of time sweep tests, where the material was subjected to constant oscillatory stress (1% strain, 1 Hz) at a physiological temperature of 37 °C for 1 h. In details, the system gelation was determined observing the sharp increase of G′ (storage modulus) compared to G″ (loss modulus) after less than 250 s. Increasing values of G′ and G″ were detected up to 1 h due to the rapid formation of collagen fibrils when the material is exposed at 37 °C, finally recording G′ and G″ values of about 275 Pa and 29 Pa, respectively (Figure 4C).

#### 3.2.2. Preliminary Printing Tests of Coll/NanoMBG_Sr4%

**Figure 5:**
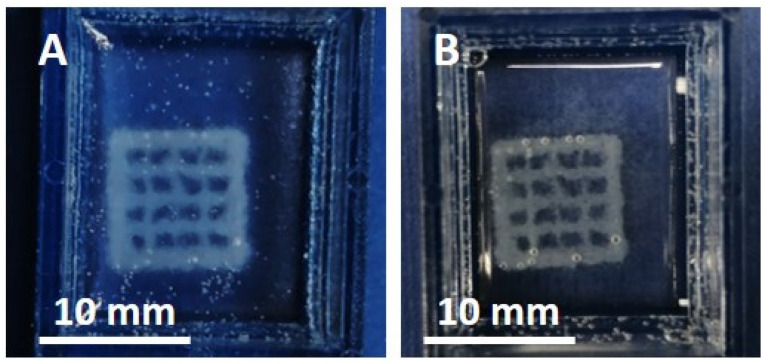
Three-dimensional printed mesh-like structure (10 × 10 × 1 mm^3^) before (**A**) and after (**B**) incubation at 37 °C.

The system printability was further assessed by preliminary tests, using an extrusion-based system equipped with 27 G needles to potentially enhance the resolution of the final 3D constructs. After loading into a 3 mL cartridge, the Coll/NanoMBG_Sr4% suspension was kept at 10 °C, to preserve its viscoelastic properties. Before printing, a gelatin slurry was poured into the multi-well system and kept at 20 °C during the entire printing process, to support the deposition of the material [29]. At this temperature, the rheological behavior exhibited by the gelatin slurry enabled the accurate moving of the needle due to the negligible mechanical resistance while holding in place the deposited material. As reported in the study of Paxton et al. [34], to obtain a filament size equal to that measured by the inner needle diameter, the pressure imparted by the printing system needs to be finely regulated to harmonize the flow rate with the printing head speed. Based on this approach, mesh-like structures (10 × 10 × 1 mm^3^) were printed at a constant pressure of 60 kPa, using a print-head translation speed of 8 mm/s. The following incubation of the scaffolds at 37 °C for 3 h promoted the sol–gel transition of the hybrid system and the removal of the gelatin extrusion bath, as shown in Figure 5. 

### 3.3. Influence of Genipin Crosslinking on the Hybrid System Properties

Considering the poor strength shown by the hybrid constructs after the collagen physical crosslinking at 37 °C, we implemented an additional crosslinking procedure. To this aim, a chemical treatment with genipin and its influence on the final properties of Coll/NanoMBG_Sr4% was optimized and investigated. In detail, after the physical crosslinking of the system at 37 °C, the use of a 0.5 wt.% genipin solution was exploited to promote the formation of inter- and intra-molecular covalent bonds. Furthermore, in order to limit the premature release of Sr^2+^ ions during the crosslinking reaction in aqueous medium, genipin in 70% EtOH was used as crosslinking solution and compared to the more traditional genipin dissolved in PBS. The resultant increase in strength and thermal stability of Coll/NanoMBG_Sr4% after crosslinking was confirmed in both the case of GEN/EtOH and GEN/PBS.

#### 3.3.1. Material Strength and Thermal Stability after Genipin Crosslinking

The successful chemical crosslinking of the hybrid collagen formulation and the resulting strength and thermal stability were assessed through rheological tests.

Based on results recently reported by the authors [39], our physical crosslinking of the collagenous system triggered by physiological-like pH and temperature led to the formation of a relatively weak structure displaying values of G′ and G″ of about 500 and 70 Pa, respectively. Moreover, the thermal stability of Coll/NanoMBG_Sr4% exposed to increasing temperatures showed that, above 46 °C, denaturation of collagen occurred due to the presence of non-covalent weak bonds between the protein molecules and the overall complex modulus of the material dropped.

**Figure 6:**
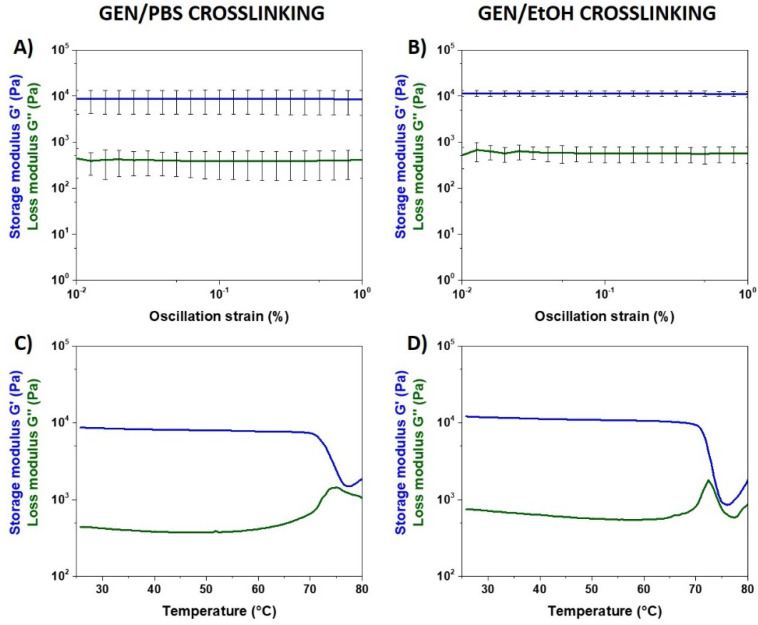
Amplitude sweep tests (**A**,**B**) and temperature ramps (**C**,**D**) performed on Coll/NanoMBG_Sr4% samples crosslinked with GEN/PBS and GEN/EtOH.

On the contrary, a significant increase in the material strength, as well as the stability at higher temperatures, was evident after both GEN/PBS and GEN/EtOH crosslinking, as clearly visible from the graphs reported in Figure 6.

Amplitude sweep tests performed at 37 °C after treating Coll/NanoMBG_Sr4% system in GEN/PBS solution gave mean values of G′ and G″ of about 8222 and 487 Pa, respectively. The hybrid formulation crosslinked by GEN/EtOH solution showed even higher strength, registering mean values of G′ and G″ of about 11,032 Pa and 599 Pa in the same range of strains. The relevant gap between G′ and G″ obtained after genipin crosslinking is indicative of a predominant elastic behavior, different from the non-crosslinked system.

Irrespective of the use of GEN/PBS or GEN/EtOH, the chemical crosslinking of collagen induced a significant increase in the material denaturation temperature, as clearly shown in Figure 6C and 6D. Genipin-crosslinked Coll/NanoMBG_Sr4% subjected to temperature ramp in the 25–80 °C range underwent a sharp decrease of the overall complex modulus of the material at about 70 °C, indicative of the structural collapse.

#### 3.3.2. In Vitro Degradation of Genipin-Crosslinked Coll/NanoMBG_Sr4%

Hydrolytic and enzymatic degradation of Coll/NanoMBG_Sr4% before and after GEN/PBS and GEN/EtOH crosslinking was investigated to further prove the stability of the system both in aqueous and enzymatic solutions.

**Figure 7:**
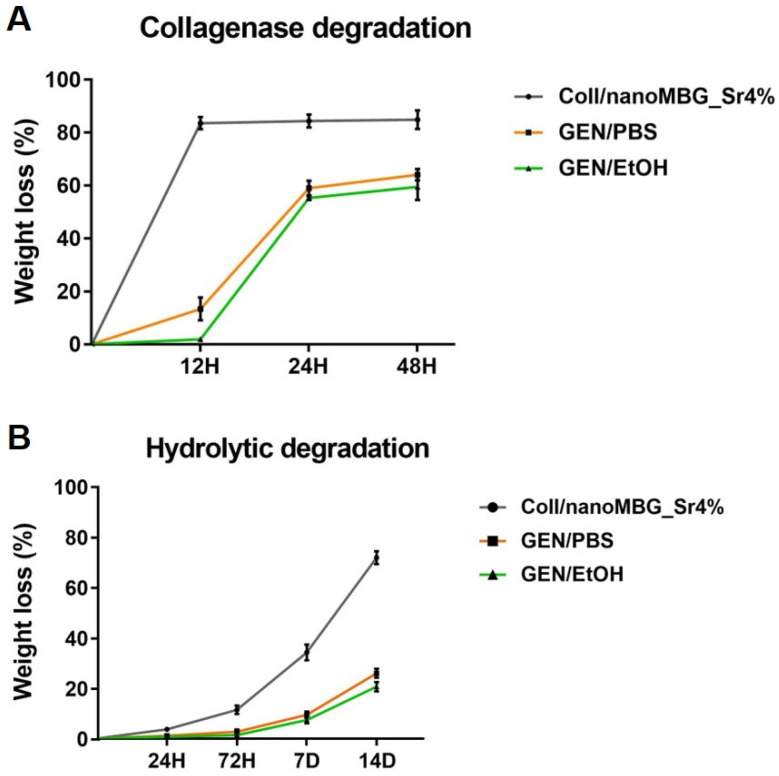
Enzymatic (**A**) and hydrolytic (**B**) degradation of the Coll/NanoMBG_Sr4% system before and after GEN/PBS and GEN/EtOH treatment.

The collagenase digestion of both crosslinked and non-crosslinked samples at 37 °C was investigated by measuring the percentage of weight loss up to 48 h of soaking in the enzymatic solution. As illustrated in Figure 7A, the degradation process of non-genipin-crosslinked Coll/NanoMBG_Sr4% samples showed a rapid weight loss, retaining less than 20% of the initial weight already after 12 h of incubation into the collagenase solution. On the contrary, both GEN/PBS and GEN/EtOH crosslinked samples retained about 40% of the initial weight after 48 h of incubation, confirming the efficacy of the chemical treatment.

In parallel, the hydrolytic degradation of Coll/NanoMBG_Sr4% before and after genipin treatment was explored up to 14 days of incubation in D-PBS at 37 °C.

Similar to the previous results, the non-crosslinked system showed less stability, retaining less than 30% of the initial weight after 14 days, as shown in Figure 7B. On the contrary, both GEN/PBS and GEN/EtOH showed high stability in the aqueous environment, registering less than 30% of weight loss at 14 days.

#### 3.3.3. Strontium Ion Release 

The release of Sr^2+^ ions from the hybrid system crosslinked with GEN/PBS and GEN/EtOH up to 14 days was analyzed by ICP. To assess the strontium released during the crosslinking treatment, the ion concentration in the supernatants collected after 10 and 24 h of Coll/NanoMBG_Sr4% contact with genipin solutions was measured. After 24 h, the crosslinked samples were transferred in Tris-HCl buffer (releasing medium) to verify the release of the remaining strontium ions. The percentage of ion released was calculated by considering the amount of strontium initially incorporated into nanoMBG_Sr4% particles (as measured by ICP on acid-digested samples) and the content of the inorganic phase in the hybrid system, assuming the homogeneous distribution of the particles.

**Figure 8:**
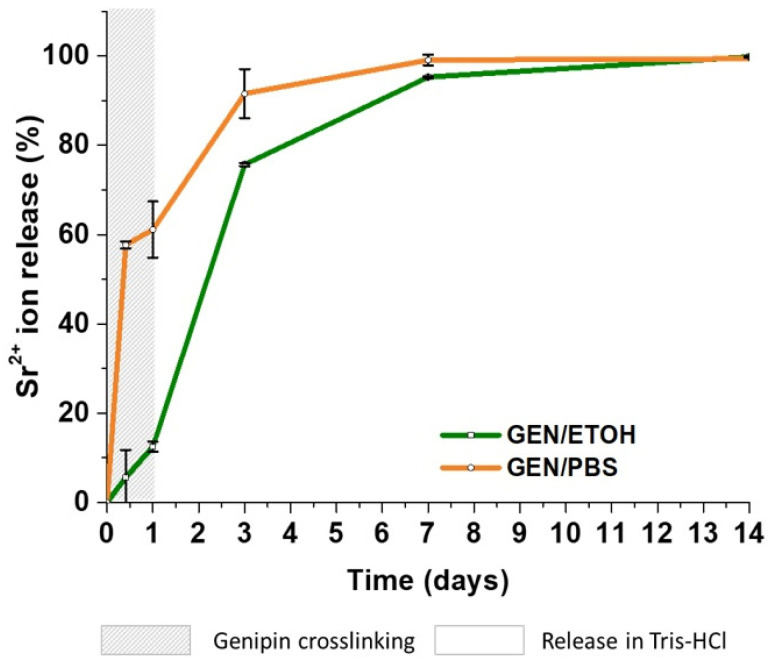
Sr^2+^ ion release from GEN/EtOH and GEN/PBS crosslinked Coll/NanoMBG_Sr4% system.

The crosslinking process caused a significant release of Sr^2+^ ions when Coll/NanoMBG_Sr4% samples were treated with GEN/PBS solution, as reported in Figure 8. In detail, GEN/PBS crosslinked samples displayed fast release kinetics reporting 57.7% and 61.1% of release, after 10 and 24 h, respectively (gray zone, Figure 8). On the contrary, the use of GEN/EtOH solution induced a significant decrease of the ion release during the crosslinking reaction time, showing a release of only 5.6% and 12.5% (gray zone, Figure 8). Once soaked in the Tris-HCl buffer, both GEN/PBS and GEN/EtOH crosslinked material samples released almost the whole strontium incorporated ions after seven days, registering values of 99.1% and 95.3%, respectively.

### 3.4. Biological Assessment

#### 3.4.1. Cell Viability

**Figure 9:**
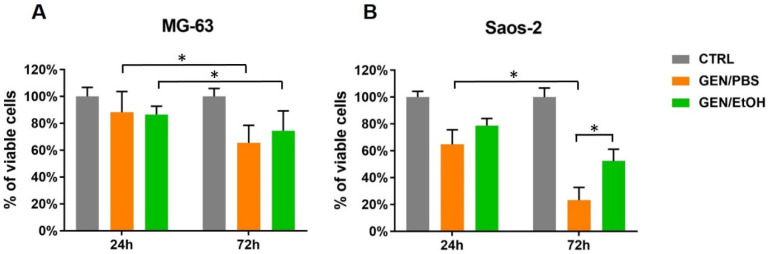
Histograms of Alamar Blue assay showing MG-63 (**A**) and Saos-2 (**B**) cell viability on GEN/PBS and GEN/EtOH crosslinked samples at 24 and 72 h (* *p*-value ≤ 0.05).

The results of cell viability (Alamar Blue assay) of MG-63 and Saos-2 cultured on GEN/PBS and GEN/EtOH crosslinked Coll/NanoMBG_Sr4% are shown in Figure 9. The background value of the biomaterial without cells, i.e., about 120 RFU, was subtracted from the RFU of cell-seeded samples. 

The results of the Alamar blue reduction confirmed the viability of MG-63 and Saos-2 on both GEN/PBS and GEN/EtOH crosslinked Coll/NanoMBG_Sr4% after 24 and 72 h of culture, with slightly higher viability found for GEN/EtOH crosslinked samples in comparison to GEN/PBS. Figure 9 clearly shows that, even if cell proliferation was not observed, cell viability was maintained after 72 h from seeding. Despite the evident decrease of Saos-2 viability on GEN/PBS crosslinked system at 72 h, no significant differences were detected between the two time points considered especially in the case of the GEN/EtOH crosslinked Coll/NanoMBG_Sr4%, confirming a cell viability preservation. Control of MG-63 seeded onto standard TCPS (CTRL) and analyzed at 24 h (907.22 RFU) and 72 h (942.33 RFU) were taken as 100% of viable cells to calculate the percent viability of the other samples. 

#### 3.4.2. Morphological Analyses

Cell adhesion and morphology of osteoblast-like cells MG-63 and Saos-2 on both GEN/EtOH and GEN/PBS crosslinked hybrid systems were assessed by SEM. Morphological features of control MG-63 and Saos-2 cells seeded onto standard TCPS are shown in Supplementary Materials Figure S3.

**Figure 10:**
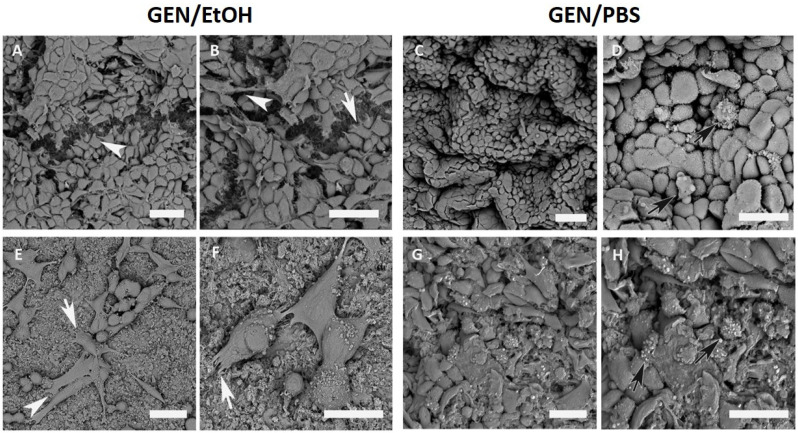
SEM micrographs of MG-63 adhesion at 24 h on 0.5% GEN/EtOH (**A**,**B**) and 0.5% GEN/PBS (**C**,**D**) crosslinked system; Saos-2 adhesion at 24 h on 0.5% GEN/EtOH (**E**,**F**) and 0.5% GEN/PBS (**G**,**H**) crosslinked system. Pointed arrows indicate cell–cell contacts, white arrows indicate cell–material interactions and black arrows show suffering features; scale bars 50 µm.

In detail, at 24 h of culture a high number of MG-63 cells adhered to the surface of both GEN/EtOH and GEN/PBS treated samples (Figure 10). On GEN/EtOH crosslinked samples, MG-63 cells appeared flat and more stretched, and contacts among neighboring cells were evident (Figure 10A,B). On GEN/PBS samples, few points of contact among cells and some round-shaped cells were observed (Figure 10C,D). Saos-2 cells displayed both round and spread shapes, with several membrane contacts with the material on GEN/ETOH treated samples (Figure 10E,F), while an unusual morphology and evident suffering features with few cell–scaffold connections were observed on GEN/PBS crosslinked system (Figure 10G,H).

**Figure 11:**
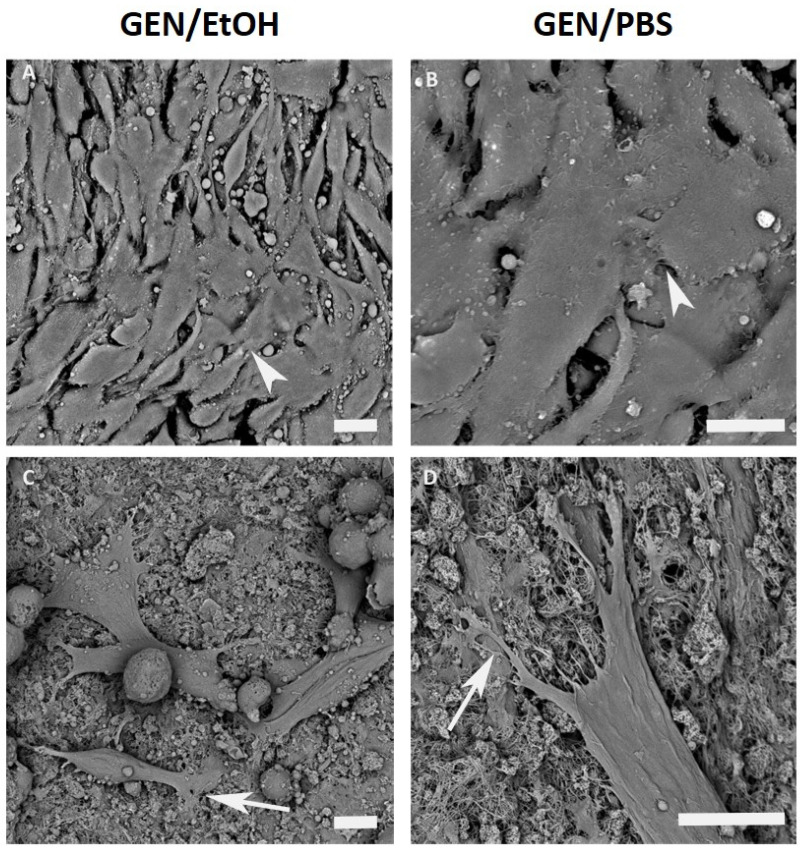
SEM micrographs of MG-63 (**A**,**B**) and Saos-2 (**C**,**D**) adhesion on 0.5% GEN/EtOH crosslinked system at 72 h. Pointed arrows indicate cell–cell contacts and white arrows indicate cell–material interactions; scale bars 10 µm.

At 72 h, MG-63 cells were still strongly adherent to the GEN/EtOH crosslinked system, showing some cell stretching and branched processes, such as filopodia and membrane protrusions (Figure 11). Similarly, Saos-2 cells displayed fusiform and star-like morphologies and multiple extensions such as lamellipodia and filopodia (Figure 11).

## 4. Discussion

The combination of type I collagen with bioactive glasses has been widely reported and is considered promising in the design of bioactive systems for BTE. Nano-sized MBG particles enriched with strontium ions largely proved their high bioactive and pro-osteogenic effect, enabling the formation of biomimetic nanocomposites when successfully embedded in fibrous collagenous matrices [20,46]. In the present contribution, to expand the encouraging results of recent studies by the authors on the combination of type I collagen with nanoMBG_Sr4% [39], the biocompatibility, bioactive properties and the potential of the developed hybrid formulation as material ink for the 3D printing of bone-like scaffolds were investigated. In addition, an effective crosslinking strategy based on genipin (a naturally derived biocompatible chemical crosslinker) was fully optimized to increase the poor mechanical properties and the premature release of pro-osteogenic strontium ions from the composite, thus maintaining MBG pro-osteogenic properties.

Firstly, FESEM was used to investigate the micro- and nano-architectures of the hybrid system after physical crosslinking at 37 °C. FESEM images proved that the presence of the inorganic phase in the hybrid formulation did not hinder the physiological self-assembly of collagen, leading to final well-packed nano-fibril matrices. Moreover, the morphological analyses clearly showed the homogeneous distribution of nanoMBG_Sr4% throughout the polymeric matrix, despite the occasional formation of agglomerates, most likely due to particle aggregation within the starting suspension. In some regions, better dispersion of nanoMBG_Sr4% promoted their arrangement along collagen fibrils, nicely resembling the arrangement of the mineral phase within the native bone matrix. This suggests that advanced biomimetic structures may potentially be achieved by improving the particle dispersion within the starting suspension. The creation of biomimetic nanostructured systems has proved to beneficially influence not only stem cells osteogenic differentiation but also to accelerate the mineralization kinetics in vivo [47,48,49,50]. Besides the formation of biomimetic nano-morphologies, one of the most important aspects of BTE is the biomineralization of the ECM [51,52]. The in vitro bioactivity of MBG is related to their ability to stimulate the formation of calcium phosphate nano-crystals when in contact with physiological fluids [7,20,48,53]. In this regard, FESEM microscopy and XRD analyses successfully confirmed the remarkable bioactive behavior of the developed hybrid formulation, evidencing the precipitation of HA crystals, especially at the surface of inorganic particles after incubation in SBF. The significant bioactive properties can be ascribed to the presence of both collagen and nanoMBG_Sr4%, as already reported by other studies [20,48]. In detail, the release of silicic acid due to MBG dissolution and the surface hydroxyl groups can induce apatite formation through ion-exchange with calcium and phosphate ions of the soaking medium. At the same time, the carboxylic groups of collagen can easily bind calcium ions, providing nucleation sites for the further deposition of calcium phosphate without altering the triple helix structure [53]. The deposition of HA crystals further boosts the biomimicry of the system, producing a final composite that contains both the main organic and inorganic phase of the natural bone extracellular matrix.

After proving the retention of the bioactive properties, the suitability of the developed formulation for 3D extrusion printing technology was investigated.

As widely reported in the literature, the rheological properties define the material printability and strongly influence the printing parameters to generate scaffolds featuring high shape fidelity depending on the selected fabrication process [29,32,34]. The presence of suspended inorganic phases may strongly affect the viscoelastic properties of the system, potentially hindering its final printability, especially when using small needles.

Flow ramp tests performed at 10 °C on the hybrid suspension demonstrated a notable decrease in viscosity in response to increasing shear rates and the presence of yield stress that are both recognized to be essential ink properties for extrusion printing technologies and typical of shear-thinning fluids [34,54,55]. 

Besides the shear rate-dependent behavior, the printability is strongly influenced by the material crosslinking and gelation kinetics upon specific conditions. The sol-to-gel transition of the hybrid system at physiological temperature (37 °C) was thus investigated to define the change in material viscoelastic properties against temperature increase. Although the sol–gel transition occurred after less than 250 s, longer times were required by Coll/NanoMBG_Sr4% to achieve a stable solid gel, corresponding to a relevant enhancement in the storage modulus (G′) values. The relatively slow sol–gel transition can be likely ascribed to the presence of nanoMBG_Sr4%, whose surface extremely rich in OH-groups can lead to abundant electrostatic interactions with amino and carboxylic residues of collagen molecules, hampering their self-assembly and packing. Likewise, Pontremoli et al. [44] also reported delayed gelation times for MBG-containing polyurethane-based systems compared to the polymeric system alone.

To further prove the potential of the developed hybrid system for 3D printing applications, we used an extrusion-based system to manufacture mesh-like structures. Among the available additive manufacturing technologies, extrusion-based systems enable the use of a wider range of suspension viscosity while providing precise control over the material deposition [34,55,56,57].

Despite the promising viscoelastic properties exhibited by the Coll/NanoMBG_Sr4% formulation, self-supporting materials for extrusion printing applications normally exhibit higher values of viscosity and yield stress, along with faster gelation and crosslinking kinetics [34,57]. Consequently, to support the partially consolidated extruded structures while preventing their collapse, we printed Coll/NanoMBG_Sr4% suspension by exploiting an innovative strategy defined as freeform reversible embedding of suspended hydrogels (FRESH) reported in recent studies [29,58]. This approach requires the use of a thermo-reversible biocompatible bath able to support the material ink deposition during the printing process, enabling the design of complex structures with improved resolution and printing fidelity. In the case of collagenous inks, a gelatin-based bath can be used to allow the simultaneous gelation of the 3D structure and the melting of the supporting material by simply raising the temperature to 37 °C after printing.

The diameter of the extruded filaments and, consequently, the final resolution obtained are strictly related to the needle diameter selected, as well as the set print-head speed and the imposed flow rates, which in turn depend on pressure and material rheological properties [34]. In this study, the selected mesh-like framework easily allowed the evaluation of shape fidelity of the 3D-printed constructs, providing strong evidence of the system printability. In particular, the proper choice of the printing parameters led to the deposition of strands of about 200 μm, using 27 G needles with an internal nozzle diameter between 190 and 210 μm. Therefore, our preliminary printing tests proved the suitability of the hybrid system for 3D-printing technologies. However, despite the positive viscoelastic properties and the good printability of the system, the poor construct stability after removal of the gelatin bath and the observed structure imperfections suggest a further refinement of the printing process. Based on these preliminary results and considering further improvements of the printing process, we can conclude that the developed hybrid system shows good potential for the design of high-resolution scaffolds, closely mimicking the complex structure of bone.

With this perspective, the design of collagen-based scaffolds intended for hard tissue regeneration requires the use of cytocompatible crosslinking treatments able to significantly reduce their overall mechanical weakness. Furthermore, scaffolds characterized by stiffness similar to that of native tissue have already proved to encourage the cell osteogenic differentiation according to the mechanotransduction pathways [16,46,51]. For this reason, the stabilization of the collagenous matrices is often achieved by the use of crosslinking agents yielding inter- and intra-molecular covalent bonds [16,59]. The use of genipin as collagen crosslinker has been widely reported to improve both its stiffness and the thermal response, also showing lower toxicity levels compared to alternative and more common crosslinking agents [16,17,59,60]. According to Kim et al. [42], genipin causes crosslinking of free amino groups of collagen through the formation of cyclic structures, which act as intra- and inter-molecular bridges along the fibers. In the present work, the genipin-based crosslinking approach was thus exploited to improve the overall stiffness and stability of the hybrid system in view of its effective use as bone scaffolds. 

A large number of studies considering the use of genipin as a collagen crosslinker usually reports the dissolution of the natural agent in aqueous solutions and the subsequent incubation of the material, where longer times and a physiological temperature of 37 °C demonstrated the best results in terms of crosslinking efficiency [16,27,61,62,63].

Starting from these data, and considering the high solubility of genipin in ethanol, we decided to compare the use of GEN/PBS and GEN/EtOH for the chemical crosslinking of Coll/NanoMBG_Sr4% to reduce the premature loss of strontium ions in PBS aqueous medium, as well as investigating the potential differences in the mechanical and biological behavior after the two treatments. 

The successful chemical crosslinking of Coll/NanoMBG_Sr4% was confirmed by the significant increase of the related viscoelastic properties, as well as the enhanced thermal stability both in the case of GEN/PBS and GEN/EtOH treatment. The important increase of the storage modulus values, in addition to the wide gap between the storage modulus and the loss modulus, proved the evident prevailing of the elastic response, leading to the formation of fully developed solid structures. Furthermore, the chemical crosslinking strongly influenced the stability of the complex fibrillar structure of collagen, as resulted from the temperature ramp tests. In detail, the denaturation temperature of collagen related to the helix-to-coil-transition of fibrils strongly increased up to 70 °C after the genipin treatment, leading to enhanced thermo-responsive properties of the hybrid system. Even if relevant differences were not registered in terms of denaturation temperature, the treatment with GEN/EtOH led to greater values of the overall complex modulus of the material, achieving higher stiffness compared to the GEN/PBS crosslinked samples, potentially due to the partial material dehydration and the consequent reduced water content.

In addition, the fibrillar nature of the collagenous matrix, as well as the good distribution of the inorganic phase, was preserved also after genipin treatment, as illustrated in Supplementary Materials Figure S2.

The successful chemical crosslinking and the stability of the genipin-treated system was further investigated in terms of hydrolytic and enzymatic degradation.

During the enzymatic degradation, collagenase cleaves two of three helical chains of collagen in specific non-polar regions, resulting in the final fibril disintegration [15,46]. The rapid weight loss of non-crosslinked samples reported in Figure 7 results from the weak intermolecular interactions between collagen chains. On the contrary, the formation of covalent bonds upon genipin treatment was confirmed by the observed gradual weight loss for both GEN/PBS and GEN/EtOH crosslinked Coll/NanoMBG_Sr4% samples, due to a slower degradation of crosslinked structures. 

The formation of stronger chemical bonds between collagen molecules was also confirmed looking at the stability of the system in aqueous solutions, where GEN/PBS and GEN/EtOH Coll/NanoMBG_Sr4% samples showed a moderate weight loss only after 14 days of incubation, in contrast with the non-crosslinked system. 

Finally, as the release of strontium ions is key to enhance the pro-osteogenic properties, promoting new bone formation in vivo [21,23,24], the ability of nanoMBG_Sr4% particles to release Sr^2+^ when embedded within collagenous structures crosslinked either by GEN/PBS or GEN/EtOH solutions was assessed. In particular, since the crosslinking treatment in saline aqueous medium can lead to the premature ion release from the hybrid system, the released Sr^2+^ concentration was monitored for the first 24 h into the GEN/PBS or GEN/EtOH solutions and then in Tris-HCl up to 14 days. 

Release profiles for Coll/NanoMBG_Sr4% samples crosslinked with GEN/PBS showed a rather high percentage (57.7% and 61.1% after 10 and 24 h, respectively) of strontium ions released in the collected crosslinking solution after 24 h and the remaining amount totally released after incubation in Tris-HCl within seven days. At variance, the use of GEN/EtOH led to a significant reduction of released ions during the crosslinking reaction time, registering significantly lower percentages equal to 5.6% and 12.5% after 10 and 24 h, respectively, overall showing a slower release kinetics up to seven days, probably thanks to the reduced water content of the crosslinked system. 

We thus proved the ability of the developed system to release strontium ions and the significant difference in terms of ion loss during the two crosslinking treatments. Considering that the chemical crosslinking represents the last step of the scaffold manufacturing process, it is fundamental to minimize the ion loss, guaranteeing almost the total release once the construct is incubated with cells.

Considering the biological assessment of the crosslinked hybrid system, GEN/PBS- and GEN/EtOH-treated Coll/NanoMBG_Sr4% samples showed similar results at 24 h for both MG-63 and Saos-2 cell types. At 72 h, Saos-2 reduced their viability on GEN/PBS crosslinked Coll/NanoMBG_Sr4%, whilst they maintained almost similar values on samples treated with GEN/EtOH, although a reduction not statistically significant was observed. A similar trend was observed for MG-63 cells. Overall, these results indicate that cell viability was maintained over time, confirming the biocompatibility of Coll/NanoMBG_Sr4%, especially when crosslinked with GEN/EtOH. Considering the results collected for the Saos-2 cells, adherent cells with a proper morphology and evident cell protrusions were observed on GEN/EtOH in comparison to GEN/PBS.

The reported data confirmed that the crosslinked samples may easily host viable cells, thus evidencing that the chemical crosslinking solutions and the overall process do not imply any relevant cytotoxic effect. 

Morphological analysis performed with SEM highlighted that all samples displayed some surface roughness, an important feature that encourages cellular adhesion, also considered an excellent property for implant integration [64]. MG-63 cells easily attached to the surface of Coll/NanoMBG_Sr4% and remained adherent for both GEN/PBS and GEN/EtOH crosslinked system samples. However, in GEN/PBS treated samples, some MG-63 cells were found to be round-shaped without evident intercellular connections, while on the GEN/EtOH crosslinked system the cells mostly showed a more flattened and stretched shape with some cytoplasmic processes extending to the close cells or the collagen surface. This different cell response can be due to differences in the mechanical and textural properties of the bioactive material subjected to the two different crosslinking treatments: The higher stiffness shown by GEN/EtOH crosslinked Coll/NanoMBG_Sr4% may, in fact, promote the attachment and spreading of cells, as confirmed in other studies [65]. In parallel, Saos-2 cells cultured on GEN/EtOH crosslinked samples showed superficial irregularities and numerous cytoplasmic processes stretched out to contact the material, as well as other cells. On Coll/NanoMBG_Sr4% samples crosslinked with GEN/EtOH cells exhibited many filopodia and lamellipodia, whose presence is important in cellular migration and scaffold invasion [66]. For both the osteoblastic-like cell types, the cell adhesion was satisfied over time, and cell–cell and cell–scaffold interactions were observed thanks to the presence of filopodia, lamellipodia and cytoplasmic processes, demonstrating the healthy state of cells and the good interactions with the material surface.

Besides the superior biocompatibility confirmed by both cell viability and morphology, the main advantage in the use of GEN/EtOH crosslinked Coll/NanoMBG_Sr4% can be mainly related to the significant reduction of strontium ion loss during the crosslinking process, enabling the release of the total amount of osteogenic ions only once the scaffold is put in contact with cells. Moreover, the reduced amount of water present in the system guaranteed higher mechanical stiffness and resistance to both hydrolytic and enzymatic degradation, further proving its potential as an effective system for the design of biomimetic bone-like scaffolds.

## 5. Conclusions

In conclusion, the developed Coll/NanoMBG_Sr4% system proved its potential as bioactive material ink for the 3D printing of nanostructured bone-like scaffolds. The high bioactivity and the ability to release pro-osteogenic Sr^2+^ ions further evidence its suitability for bone regeneration applications. The achievement of a multiscale system was confirmed by morphological analyses, where a biomimetic structure related to the arrangement of nano-sized MBG particles along the reconstituted collagen fibers was observed at the nano-scale.

This study optimized a crosslinking strategy based on cytocompatible genipin solution treatment, which considerably enhanced the physicochemical stability and viscoelastic properties of the hybrid system. In particular, the treatment with GEN/EtOH hindered the burst release of strontium ions, compared to GEN/PBS, thus potentially extending the therapeutic effect of the final construct.

The developed hybrid system largely proved its biocompatibility in presence of MG-63 and Saos-2 cells, especially after the crosslinking with GEN/EtOH, where the material composition and stiffness promoted a more positive cell response.

## Supplementary Materials

The following are available online at https://www.mdpi.com/2079-4991/10/9/1681/s1. Figure S1: FESEM image (A) and size distribution (B) of nanoMBG_Sr4% particles. Figure S2: Cross-sectional FESEM images of Coll/NanoMBG_Sr4% after GEN/EtOH crosslinking at different magnifications. Figure S3: SEM images showing the morphology of control MG-63 (left) and Saos-2 (right) onto standard TCPS at 24 h. Scale bars = 20 µm. Table S1: Summary of the formulation and chemical crosslinking investigated agents in the study.
