# Supplementary material for: Collagen Hybrid Formulations for the 3D Printing of Nanostructured Bone Scaffolds: An Optimized Genipin-Crosslinking Strategy
Source: Nanomaterials (Basel). 2020 Aug 27;10(9):1681. doi: 10.3390/nano10091681 (PMC7558137; doi:10.3390/nano10091681)
Supplement: Supplementary file 1 [file nanomaterials-10-01681-s001.pdf]

Article

Collagen hybrid formulations for the 3D printing of nanostructured bone scaffolds: an optimized genipin-crosslinking strategy

Table S1. Summary of the formulation and chemical crosslinking investigated in the study.

FORMULATION	CHEMICAL CROSSLINKING
1.5%Coll/nanoMBG_Sr4%	No chemical crosslinking
GEN/PBS crosslinked 1.5%Coll/nanoMBG_Sr4%	0.5% Genipin in PBS
GEN/EtOH crosslinked 1.5%Coll/nanoMBG_Sr4%	0.5% Genipin in 70% EtOH

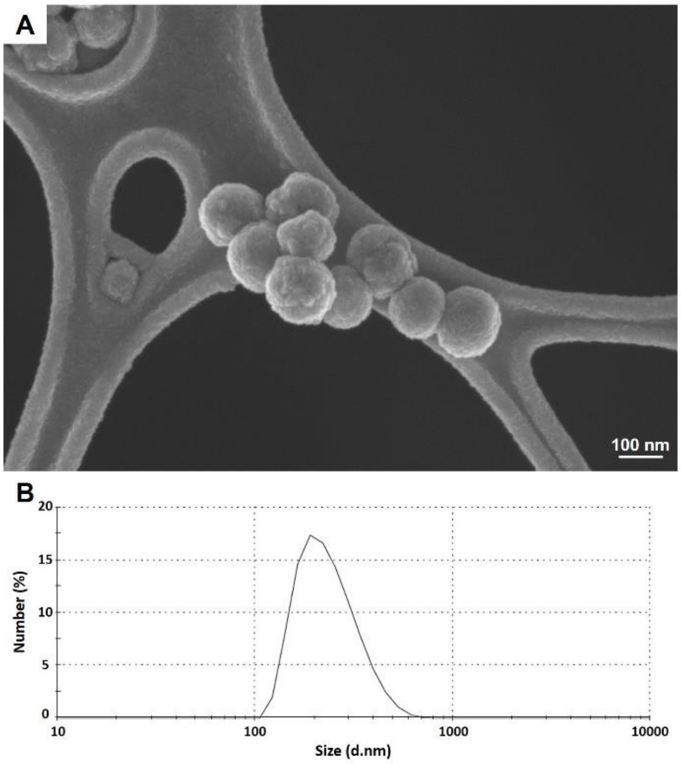

Figure S1. FESEM image (A) and size distribution (B) of nanoMBG_Sr4% particles.

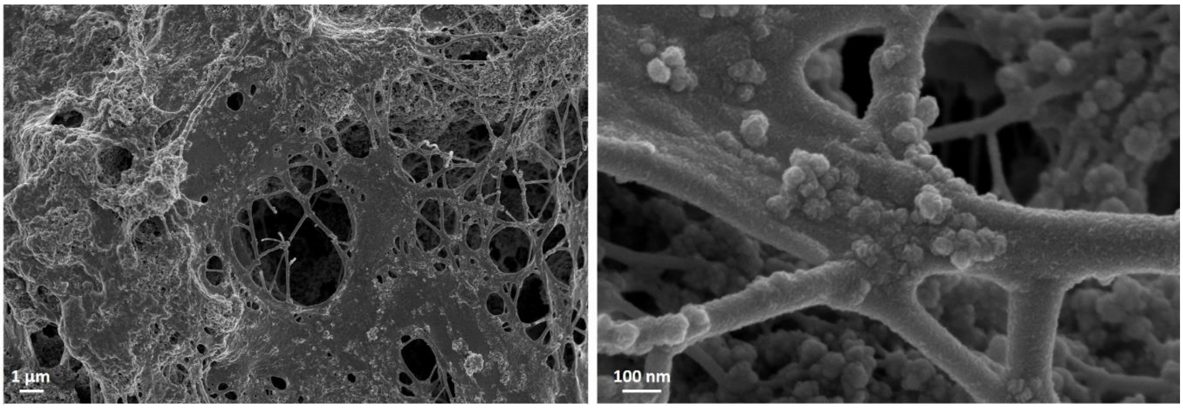

Figure S2. *Cross-sectional* FESEM images of Coll/nanoMBG_Sr4% after GEN/EtOH crosslinking at different magnifications.

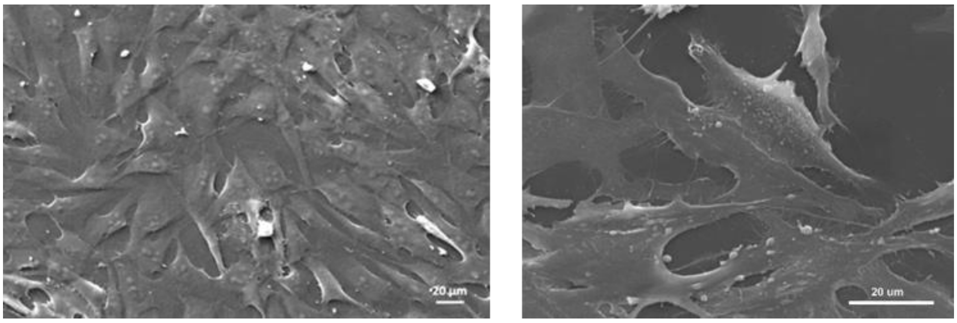

Figure S3. SEM images showing the morphology of control MG-63 (left) and Saos-2 (right) onto standard TCPS at 24 hours. Scale bars=20 μm.
